# Green and facile sol–gel synthesis of the mesoporous SiO_2_–TiO_2_ catalyst by four different activation modes†

**DOI:** 10.1039/d0ra07569h

**Published:** 2020-10-29

**Authors:** Héctor Pérez, René Miranda, Zenaida Saavedra-Leos, Ramon Zarraga, Pedro Alonso, Edgar Moctezuma, Joel Martínez

**Affiliations:** Facultad de Ciencias Químicas, Universidad Autónoma de San Luis Potosí SLP Mexico 78210 atlanta126@gmail.com; Facultad de Estudios Superiores Cuautitlán, Universidad Nacional Autónoma de México Estado de México Mexico 54740; Coordinación Académica Región Altiplano, Universidad Autónoma de San Luis Potosí Matehuala SLP Mexico 78700; Departamento de Química–DCNE, Universidad de Guanajuato Guanajuato Mexico 36050

## Abstract

The most environmentally friendly protocol for obtaining mesoporous SiO_2_–TiO_2_ catalysts has been sought. Water has been employed as a green solvent, the energy input has been minimized, and three further principles (1, 3, and 12) of Green Chemistry have been considered. Four different modes for promoting the reaction have been comparatively evaluated, namely near-infrared and microwave electromagnetic irradiations, ultrasound, and traditional mantle heating. Brunauer–Emmett–Teller (BET) analyses of the catalysts produced revealed that the non-conventional activation modes afforded both large surface areas (335–441 m^2^ g^−1^) and smaller crystal sizes (7.2–15.3 nm) than the mantle heating process. These modes also generated the catalysts in shorter reaction times than traditional mantle heating, 10–30 min *versus* 3 h, with anatase as the sole crystalline phase. The photocatalytic degradation of 4-chlorophenol has been carried out to assess the catalytic efficiencies of the hybrid materials. The catalyst synthesized with microwave assistance showed the best mineralization activity (97%), followed by those prepared with ultrasound, near-infrared, and mantle heating. The materials have been extensively characterized by FTIR, XRD, DRS-UV/Vis, SEM, ^29^Si MAS NMR, and BET analyses. To the best of our knowledge, this is the first such comparative assessment of green energetic alternatives in developing a sol–gel process.

## Abstract 2

The most environmentally friendly protocol for obtaining mesoporous SiO_2_–TiO_2_ catalysts has been sought.

## Graphical abstract

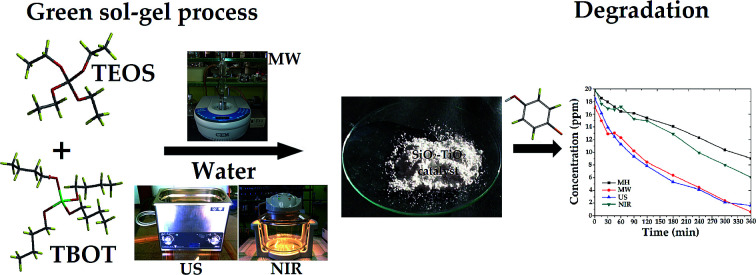

## Introduction

The sol–gel process is one of the most prevalent routes for producing oxide materials with control of their textural and surface properties, furnishing them with high purity and homogeneity.^1–3^ Due to their mesoporous structures, oxide materials are important in many industrial processes, for example as catalysts,^4^ absorbents,^5^ gas sensors,^6^ and photocatalysts.^7^ Established methods involve both the hydrolysis and polycondensation of metal alkoxides, producing homogeneous oxides at lower temperatures.^3^ The SiO_2_–TiO_2_ system has interesting applications as a photocatalyst, facilitating the elimination of several pollutants from water.^7^

Previously reported approaches for the production of SiO_2_–TiO_2_ hybrid catalysts are associated with several eco-disadvantages, arising from the use of slightly toxic organic or hazardous inorganic acids, such as HOAc,^8–10^ HCl and H_2_SO_4_,^9^ or HF,^11^ and the requirement for auxiliary substances, such as 2-methoxyethanol,^2^ ethanol,^8^*n*-propanol,^12^ and ammonia solution.^10^ Moreover, these procedures generally involve long reaction times, in addition to extended drying and aging times,^8–12^ and sometimes require supercritical conditions.^10^

On the other hand, Green Chemistry is a working paradigm at the molecular level aimed at sustainability;^13^ this guiding mode to chemical production has a protocol of Twelve Principles.^14^ In this regard, researchers strive to design chemicals and chemical manufacturing processes that pose insignificant risk to human health and the environment, although it must be conceded that no activity is risk-free.^15^

As part of our research program, we are interested in the implementation of green procedures, mainly using non-conventional activation methods, such as near-infrared (NIR) and microwave (MW) irradiations or ultrasound (US).^16^ Consequently, the goal of this work is the achievement of a green approach^17^ for the production of SiO_2_–TiO_2_ catalysts, comparing four different modes for activating the reaction, namely NIR, MW, US, and traditional mantle heating (MH), applying various principles of the Green Chemistry protocol. To the best of our knowledge, this is the first report comparing the efficacies of these activation modes in generating the target materials.

## Results and discussion

### Fourier-transform infrared spectroscopy (FTIR)

**Fig. 1:**
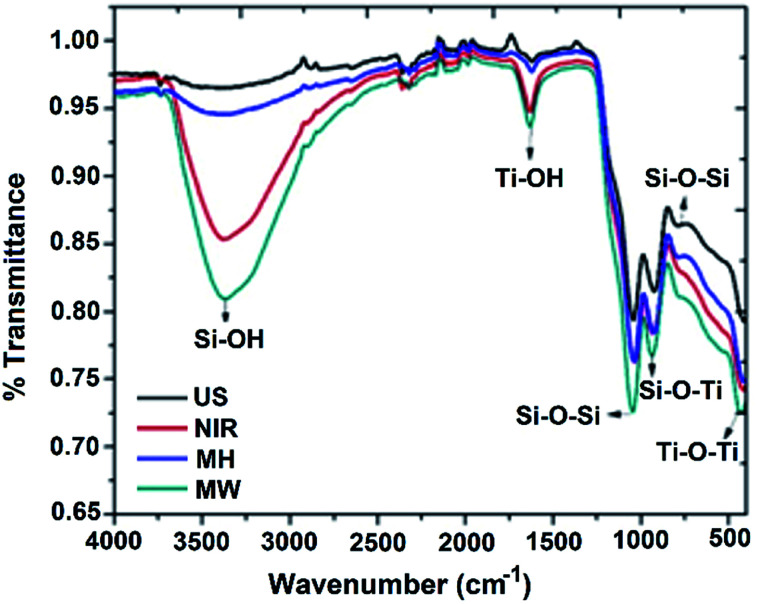
FTIR spectra of SiO_2_–TiO_2_ (1 : 1) catalyst obtained by MH, US, NIR, and MW.

The infrared spectra of the target materials, taking the 1 : 1 molar ratio catalysts as representative examples, are presented in Fig. 1. Data for the other molar compositions are summarized in Table S1.†

The bands in the ranges 1057–1039 and 806–795 cm^−1^ can be assigned to symmetric and asymmetric stretching vibrations, respectively, of Si–O–Si bonds. Bands at 931–919 cm^−1^ can be attributed to a stretching vibration of Si–O–Ti bonds, and the broad bands in the range 432–410 cm^−1^ can be assigned to symmetric stretching vibrations of Ti–O–Ti units. Intense broad bands in the range 3379–3326 cm^−1^ correspond to the hydroxyl group of the silanol moiety and, complementarily, bands in the range 1636–1617 cm^−1^ can be attributed to a bending mode of this hydroxyl group.

It should be borne in mind that titania strongly retains adsorbed undissociated water due to the strong Lewis acidity of its coordinatively unsaturated Ti^4+^ surface sites.^18^ Considering the intensities of the bands assigned to the Si–OH moiety (3379–3326 cm^−1^) in Fig. 1, an intense broad band is seen for the sample prepared by the MW method, indicating a catalyst with higher surface area and higher titania content in comparison to those prepared with NIR, US, or MH assistance, respectively. The bands of lower intensity may be explained in terms of a deficit of titania on the surfaces of the materials. Considering a 1 : 4 catalyst molar ratio for all four methods (Fig. S1a†), the principal differences were observed for the broad and intense Ti–O–Ti band. In addition, the Si–OH band was more intense for the samples prepared with MW and NIR assistance; less intense bands for those prepared with US and MH were probably due to a lower titania content, and hence, less retention of undissociated water. For a 4 : 1 catalyst molar ratio (Fig. S1b†), the Ti–O–Ti band was less well-defined for all of the samples. The Si–OH bands were slightly less intense compared to the other molar ratios, probably because the higher silica content and lower titania content resulted in less undissociated water on the surface. In summary, the FTIR spectral data are consistent with the proposed compositions of the SiO_2_–TiO_2_ mixed-oxide catalysts.

### X-ray diffraction (XRD)

**Fig. 2:**
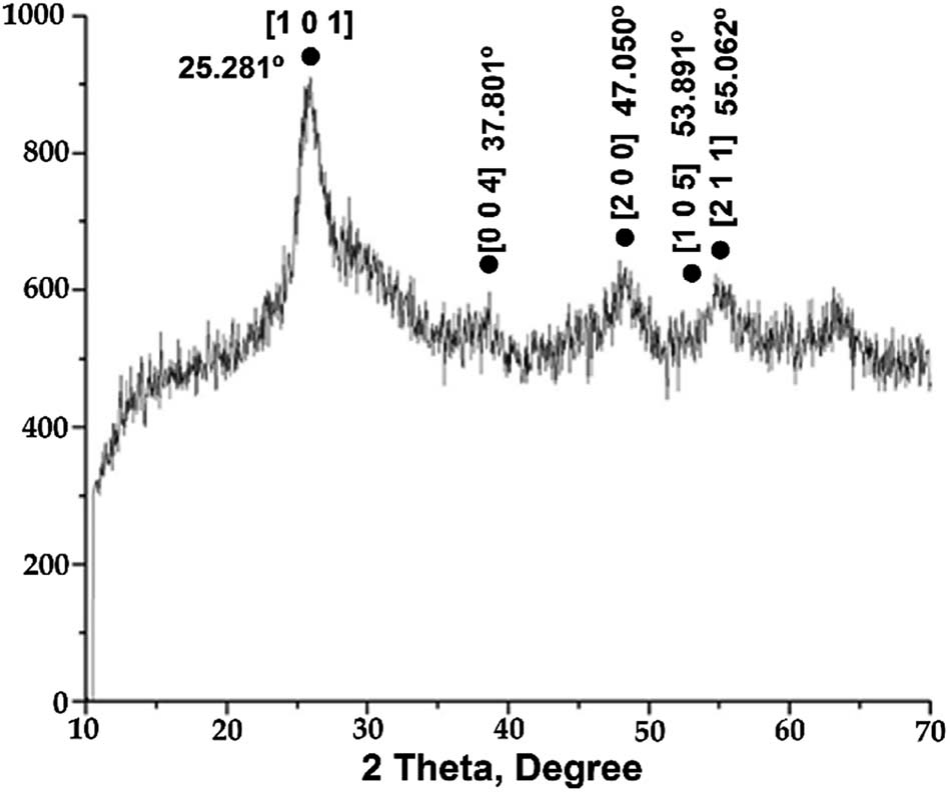
Characteristics peaks of TiO_2_ from (1 : 4) SiO_2_–TiO_2_ catalyst.

The diffraction patterns of the prepared catalysts reflect their amorphous nature. The expected signals of the silica component were not visible, implying that it exists in an amorphous state.^19^ Moreover, the signals of the anatase TiO_2_ crystalline phase were not well defined due to the high content of silica in the network, causing signal overlap (Fig. 2). Greater intensity of the peaks assigned to the anatase phase indicates that more titania is deposited on the silica surface.^11^ For the 4 : 1 and 1 : 1 SiO_2_–TiO_2_ molar ratios (Fig. S2†), the diffraction patterns were similar. That is to say, because of the amorphous nature of the silica, it was difficult to detect the diffraction peaks of titania due to signal overlap. Specifically, at the 1 : 1 molar ratio, the peaks at 25.281 and 47.050° were at their least intense (Fig. S2a†); at the 4 : 1 molar ratio, only a very broad diffraction peak at 25.281° could be discerned.

Evidently, the addition of silica significantly influences the phase transition of titania, inhibiting formation of the crystalline rutile phase. On the other hand, the calcination process improves the crystallinity of the sample. Thus, the obtained results imply a better atomic structure between TiO_2_ and SiO_2_, inhibiting the rutile phase transformation and increasing polycrystallization. This may be ascribed to the formation of strong Ti–O–Si bonds, diminishing the crystal size. Consequently, these materials have high thermal stability, allowing their calcination at 500 °C without formation of the rutile phase.^20^

### Differential reflectance spectroscopy-UV/Vis (DRS-UV/Vis)

According to the DRS-UV/Vis spectral data summarized in Table S2,† the absorption edge of SiO_2_–TiO_2_ appears in the UV region. Evidently, the wavelength values were not noticeably modified in comparison to that for TiO_2_ (3.2 eV).

**Fig. 3:**
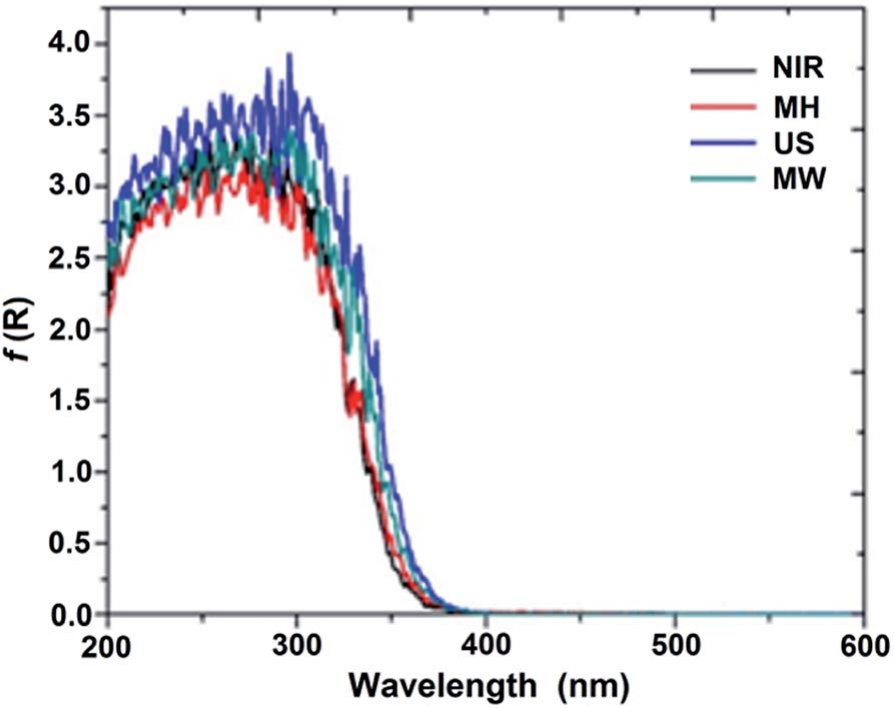
Curves of Kubelka–Munk of SiO_2_–TiO_2_ (1 : 1) catalyst.

Band gaps for all of the SiO_2_–TiO_2_ hybrid catalysts were calculated by the Kubelka–Munk^21^ method (Fig. 3), and proved to be proportional to the absorptions of the samples.^8^ The obtained results illustrate that the shift in the absorption edge for the SiO_2_–TiO_2_ catalyst depends on the synthetic method, the nature of the precursors, and the calcination temperature of the sample.^22^

According to the above observations, the catalysts prepared with MW and US assistance show slightly stronger absorptions, and hence smaller band gaps, compared to those prepared by the NIR and MH methods. This absorption may be associated with the charge-transfer transition between the constituent atoms titania and the higher content of titania on the surface of the network.^8^ Good mineralization of 4-chlorophenol is achieved due to the convenient crystal size of the composites; see below. As shown in Fig. S3a,† the catalyst with a 1 : 4 molar ratio showed similar behavior to that with a 1 : 1 molar ratio. For the catalyst with a 4 : 1 molar ratio, higher absorption of the US was observed, due to the greater charge-transfer transition between the atoms of titania, possibly due to small spherical agglomerates thereof. The band gaps were higher than that for TiO_2_.

### Brunauer–Emmett–Teller analysis (BET)

The adsorption–desorption isotherms of the SiO_2_–TiO_2_ catalysts were seen to be of type IV, characteristic of agglomerated mesoporous materials, according to the Brunauer–Demming–Demming–Teller (BDDT) classification.^23^ According to IUPAC classification, a clear H3-type hysteresis loop was detected, which is related to the occurrence of pore condensation. The first part of the sorption isotherm is associated with weak interactions between the adsorbent and the adsorbate. An H3-type hysteresis loop is the most common for inorganic oxides, often associated with a mesoporous material consisting of channels of cylindrical pores or agglomerates of non-uniform spherical particles.^24^

The specific surface areas calculated using the multi-point BET method, the average pore diameters (Barrett–Joyner–Halenda (BJH) method), and the total pore volumes are provided in Table 1. The differences in crystal sizes and surface areas between the samples can be explained by considering the production modes, since the extent of polymerization is dependent on the number of moles of water added to the alkoxide. An increase in H_2_O/alkoxide ratio increased the BET surface area. The extent of cross-linking determines the degree of polymerization.^25^ Accordingly, a higher H_2_O/alkoxide ratio was used, resulting in smaller crystal sizes and a higher BET surface area.

**Table 1:** Physical properties calculated by mean BET and BJH methods for SiO_2_–TiO_2_ (1 : 1) catalyst

Properties	NIR	MW	US	MH
Area (m^2^ g^−1^)	335	441	417	408
Pore size (nm)	4.33	5.57	4.78	2.77
Pore volume (cm^3^ g^−1^)	0.363	0.585	0.527	0.290
Crystal size (nm)	15.3	7.2	7.9	8.5
Mineralization (%)	70	97	91	54

The best physical properties were obtained by the MW procedure, which may be attributed to better physical behavior of the mixture reaction in terms of mass transfer and diffusion. Inferior physical properties were obtained by the MH method, due to slow diffusion and mass transfer in the reaction mixture. For the NIR method, the surface area was probably reduced by partial evaporation of water from the reaction mixture.

### Chemical synthesis

As mentioned above, after an extensive search of the literature,^12,13,26,27^ we believe this to be the first report concerning comparison of the various activation methods in the production of such mixed-oxide catalysts.

Regarding the use of MH, it should be borne in mind that heat is carried out by conduction from the source to the reaction mixture. Consequently, a long reaction time is required for the hydrolysis. Moreover, the obtained composites have good surface area, but small particle size and low pore volume.

For activation by NIR radiation, it must be taken into account that this mode induces vibrations of the chemical bonds of the molecules (stretching, bending, rocking, and twisting), matching the absorption characteristics of both the solvent and the reagents. Thus, NIR is absorbed by the mixture, producing the essential heat to promote the reaction.^13^ This process provided the target catalysts with lower surface area (335 m^2^ g^−1^). Consequently, the hydrolysis process was affected; however, both the pore diameter and volume were improved in comparison to the MH process.

As regards US activation of the reaction, the obtained results can only be attributed to cavitation effects inside micro drops of solvent, decreasing the diffusion layer and mass transfer.^28^ The energy input is sufficient to initiate nucleation, leading to growth of a three-dimensional network. However, application of a mechanical force can induce breaking of the network, leading to agglomeration of the material with concomitant decreases in both surface area and pore size.

In the case of MW assistance, activation is favored by the polarity of the reaction medium, due to the capability (tan *δ*) of several compounds (mainly solvents) to transform electromagnetic energy into heat.^29^ Thus, the solvent transmits heat to the reagents, in the present case promoting hydrolysis and polymerization. In other words, the quantity of heat transferred enhances the reaction rates, generating the network in a conveniently short time, with large surface area and pores of optimal size and volume. As a general important result, in this work, the NIR and MW processes must be considered as the best options for producing this type of catalyst, because they afford the products in a shorter time than the MH method.

It is important to highlight that the abovementioned features are in full compliance with the green chemistry protocol (GCP).^14^

Firstly, the by-products of the reaction, ethanol derived from tetraethylorthosilicate (TEOS) and *n*-butanol derived from tetrabutylorthotitanate (TBOT), are both slightly toxic, but are biodegradable according to the Toxic Release Inventory of the Environmental Protection Agency (TRI-EPA).^30^ These features conform to three of the principles of the GCP: principle one (prevention), three (less hazardous chemical synthesis), and ten (degradation).

Secondly, we note that in previous reports concerning the production of SiO_2_–TiO_2_, the use of HCl, HF, or H_2_SO_4_ was described,^8–12^ all of which are highly toxic according to the TRI-EPA. In this work, these substances were avoided, conforming to two principles of the GCP: three (less hazardous chemical synthesis) and twelve (inherently safer chemistry for accident prevention). As regards principle five (safer solvents and auxiliaries), the described procedure represents a good green approach since the solvent employed, water, is considered as one of the greenest solvents. Finally, as regards principle six (design for energy efficiency), greener novel modes (NIR, US, MW) for activating the reaction are offered as better alternatives to traditional MH.

### Field-emission scanning electron microscopy (FE-SEM)

**Fig. 4:**
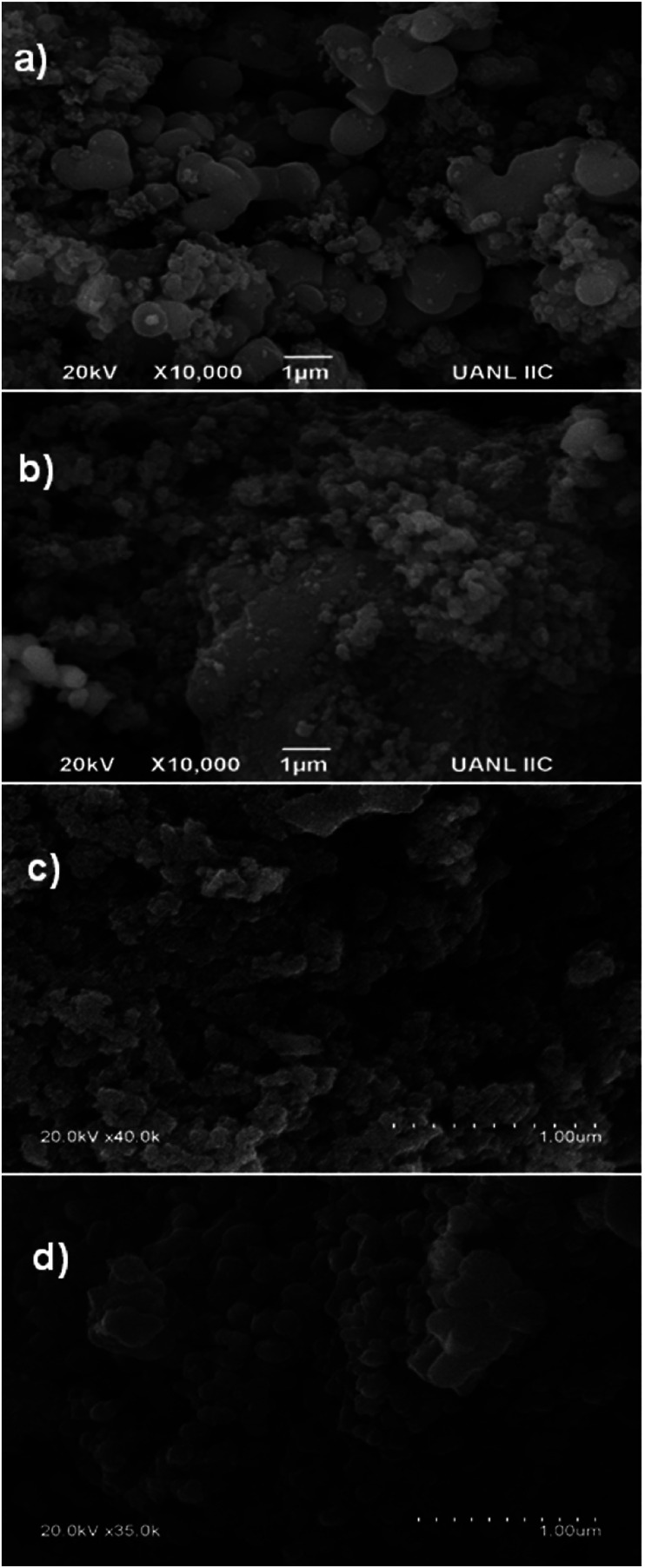
SEM micrographs of (1 : 1) molar ratio: (a) MH, (b) US, (c) NIR, and (d) MW.

As can be seen in Fig. 4a, for the sample with a 1 : 1 molar ratio, the FE-SEM image shows that the catalyst produced by means of MH was composed of uniform spherical micro aggregates of TiO_2_ with a rough external surface, together with irregular spheres of SiO_2_. The obtained low surface area (Table 1) can probably be ascribed to weak interactions between SiO_2_ and TiO_2_ and less dissolution of Si and Ti in the reaction mixture.

Concerning the employment of US as the activation procedure (Fig. 4b), a rough external surface is observed, composed of irregular spheres of SiO_2_ and small spherical agglomerates of TiO_2_. Irregular large crystals of SiO_2_ can also be observed, which probably arose from cavitation bubbles acting as nuclei and initiators of seeding.^26,28^ Moreover, collapse of the bubbles could lead to agglomeration of both kinds of particles, favoring the growth of many larger particles (*vide supra*). A poor stirring effect could delay diffusion across the layer, diminishing mass transfer.

Regarding the products from the employment of NIR and MW as activation procedures (as shown in Fig. 4c and d, respectively), they are composed of small uniform spherical particles of TiO_2_ together with more regular uniform spheres of SiO_2_. It is important to highlight that with these electromagnetic irradiations, the activation energy constitutes an excellent indicator of the improvement of the reaction.^13,29^ It has also been reported that the electric components of these irradiations exert vibrational and orientational effects, modifying the activation energy term in the Arrhenius equation and improving the process.^31^ These heating modes are fundamentally different from conventional heating since the electromagnetic radiations interact directly with the reagents, leading to efficient energy transfer at the molecular level, improving the dissolution and interaction of Si and Ti in the reaction mixture, resulting in faster formation of abundant nuclei and hence a shorter nucleation period. On the contrary, conventional heating is transmitted by conduction to the interior of the sample and is strongly dependent on efficient stirring to facilitate the distribution of heat.^29,31*b*^ In other words, with the employment of NIR and MW as activation modes, more nuclei are formed in the mixture; thus, small crystals are formed, producing aggregates composed of smaller particles.

On the other hand, thermal stability is an essential prerequisite in many potential applications of nanomaterials, but conventionally produced titania nanoparticles exhibit low thermal stability.^32,33^ By the proposed process, it is possible to achieve a large BET surface area, thereby decreasing the crystal size and the crystallization time. The XRD, SEM, and BET surface area results presented herein imply good thermal stability, which we ascribe to strong interactions between SiO_2_ and TiO_2_.

### ^29^Si magic angle spinning nuclear magnetic resonance (^29^Si MAS NMR)

**Fig. 5:**
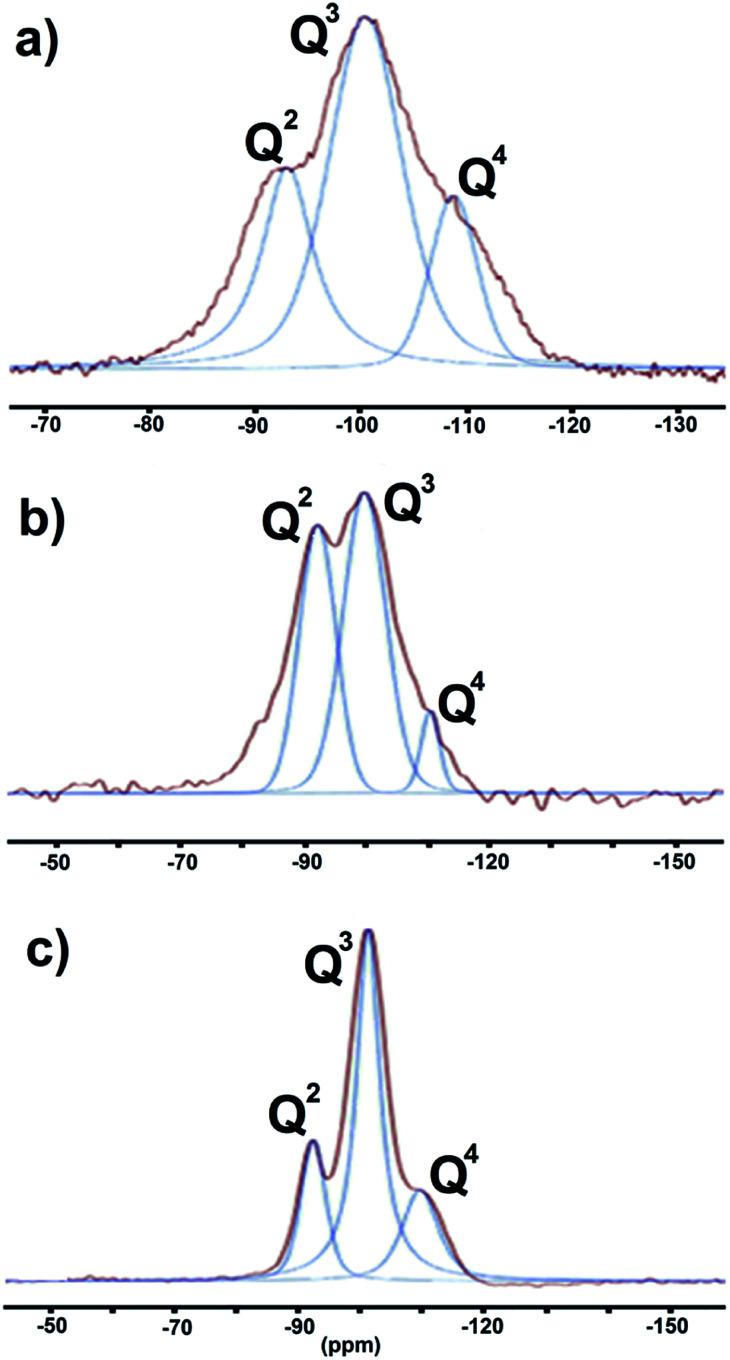
The ^29^Si MAS NMR-deconvolution spectra: (a) SiO_2_–TiO_2_ (1 : 1), (b) SiO_2_–TiO_2_ (1 : 4), (c) SiO_2_–TiO_2_ (4 : 1).

Fig. 5 shows representative ^29^Si MAS NMR for the 1 : 1, 1 : 4, and 4 : 1 composites generated with the assistance of NIR, US, and MW, respectively. After peak deconvolution, three signals were observed at *δ* = −92, −100, and −109 ppm with different intensities. Each of the signals represents a specific degree of polymerization; Q denotes a silicon atom center and the superscripts 2, 3, and 4 indicate the number of Si–O–Si bridges connected to Q. The signals at *δ* = −92, −100, and −109 ppm correspond to Si sites in Q^2^, Q^3^, and Q^4^ configurations, respectively. Thus, the respective signals correspond to one, two, or no OTi groups^35^ around each Si atom.

The proportions of the different Si species were estimated from the integrated areas of their peaks (Table 2). For the 1 : 1 catalyst, the content of Q^3^ (48%) was approximately twice those of Q^2^ (27%) and Q^4^ (23%). For the 1 : 4 catalyst, the proportions of Q^2^, Q^3^, and Q^4^ were 40%, 51%, and 8%, respectively, and for the 4 : 1 catalyst they were 19%, 61%, and 18%, respectively, indicating that Q^4^ sites accounted for only a small fraction of the Si–O–Si tetrahedra. Consequently, the majority of Si nuclei are surrounded by OTi or OH groups, implying a high degree of titanium substitution in the silica network, that is, extensive formation of Si–O–Ti and Si–OH bonds.^34^ In order to more accurately assess the number of Si–O–Si bonds, we employed the AF_Q_ parameter,^23,36^ calculated according to the following equation:
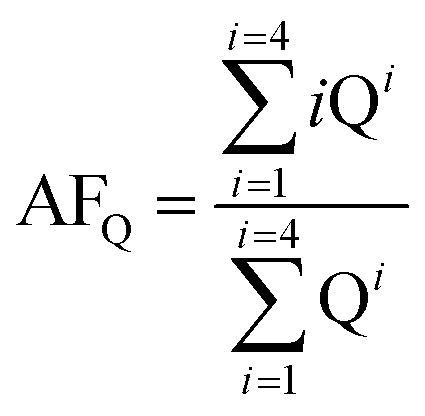
where Q^*i*^ is the integrated area of the relevant peak in the ^29^Si MAS NMR spectrum, and *i* is the number of Si–O–Si linkages about each Si atom. The 1 : 1, 1 : 4, and 4 : 1 mixtures have AF_Q_ values of 3.53, 3.00, and 3.30, respectively.

**Table 2:** Chemical shift (*δ*) in ppm, and percentage of Si structural sites

Molar ratio SiO_2_–TiO_2_	*δ*/%		
	Q^2^	Q^3^	Q^4^
1 : 1	−92.2/27.7	−100.3/48.4	−108.7/23.9
1 : 4	−92.9/40.4	−100.5/51.6	−110.3/8.0
4 : 1	−92.2/19.5	−101.3/61.8	−110.0/18.6

**Chart 1:**
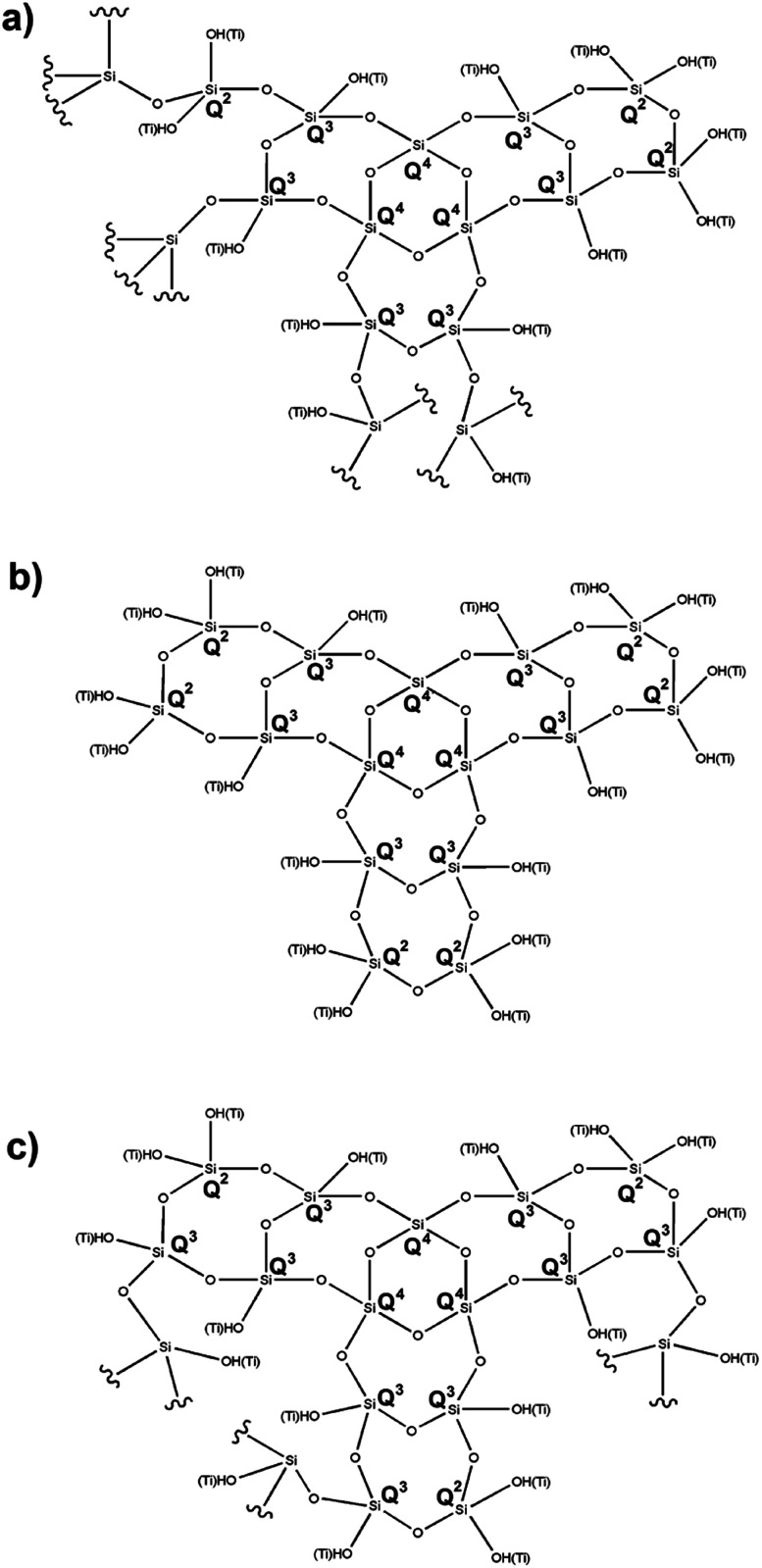
Structures proposed for SiO_2_–TiO_2_ catalyst: (a) 1 : 1, (b) 1 : 4, and (c) 4 : 1.

Based on the above data, it is possible to propose appropriate structures for the respective synthesized hybrid catalysts. Chart 1a shows the proposed structure for the 1 : 1 catalyst, maintaining the twofold greater amount of Q^3^ over the Q^2^ and Q^4^ centers. Chart 1b shows a similar ratio between Q^3^ and Q^2^ in the 4 : 1 molar ratio catalyst. Chart 1c shows a threefold greater amount of Q^3^ with respect to the Q^2^ and Q^4^ centers, bearing in mind that Q^4^ is present in a minor proportion and is considered as the center of the network. In addition, we are convinced that all TiO_2_ is incorporated into the silica network, and that the different molar ratios are not important for generating composites of this type.

### Photocatalytic assays

**Fig. 6:**
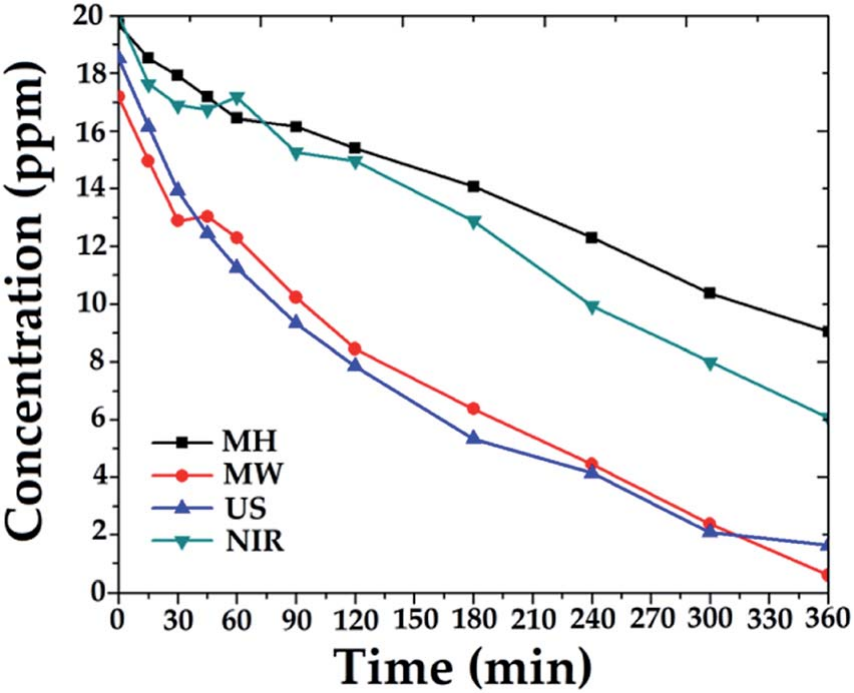
Profile of degradation of 4-chlorophenol, employing SiO_2_–TiO_2_ (1 : 4) catalysts.

The photocatalytic degradation of 4-chlorophenol was performed to determine the mineralization efficiencies of the hybrid materials.^37^ For example, 250 mg of each 1 : 4 catalyst was applied with irradiation for 6 h. Comparative degradation profiles are shown in Fig. 6.

Degradation percentages over the 1 : 4 catalysts were in the range 54–97%, depending on the activation mode. The best catalytic activity was obtained with catalysts synthesized with MW assistance, followed by US, NIR, and MH synthesis, respectively.

The obtained results are consistent with the physical properties listed in Table 1. In other words, the combination of surface area, pore size, pore volume, and crystal size of catalysts synthesized by MW favors adsorption of 4-chlorophenol molecules and facilitates their diffusion into the catalyst. Similar behavior was observed for the catalyst synthesized with US assistance; thus, advanced degradation was attained.

Comparing the other generated materials, those prepared with NIR and MH assistance both showed lower adsorptions of 4-chlorophenol, which may be ascribed to the sizes and volumes of their pores, 4.33 and 2.77 nm and 0.363 and 0.290 cm^3^ g^−1^, respectively, in this last probably due to cluster growth. Consequently, less mineralization was achieved over the sample prepared by the MH procedure.

**Fig. 7:**
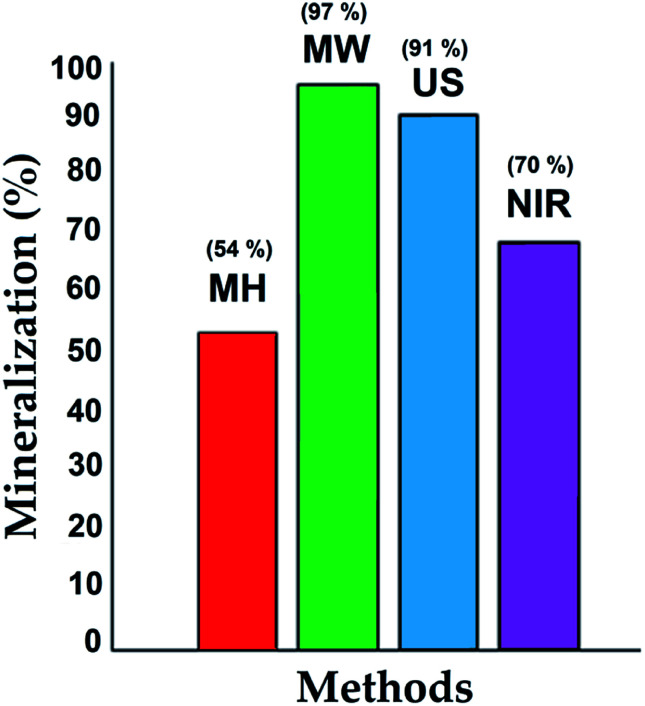
Mineralization of 4-chlorophenol, employing SiO_2_–TiO_2_ (1 : 4) catalysts.

During the photocatalytic assays, total organic carbon analyses were performed, indicating the mineralization (CO_2_ and H_2_O); the results are shown as a bar chart in Fig. 7. At the beginning of the process, a significant amount of 4-chlorophenol was adsorbed on the catalyst, and then its degradation process at the active sites (TiO_2_) proceeded under illumination with UV light.^37^ The results were consistent with the characterization of the materials, with high surface areas, appropriate pore size and volume, and small crystal size favoring adsorption of the contaminant and its interaction with the TiO_2_ incorporated in the structure of the SiO_2_. The degradation over these materials showed a synergistic effect between adsorption and photocatalytic activity.

## Experimental

### General information

Tetrabutylorthotitanate (TBOT), tetraethylorthosilicate (TEOS), were purchased from Sigma Aldrich Chemical and were used without further purification. Near-infrared irradiation was generated using a commercial device “Flavor-Time®” (1350 W/110 V/120 V–60 Hz|220 V/240 V–60 Hz). Microwave-assisted synthesis was performed using a CEM Focused Microwave™ Synthesis System, Discover model. Ultrasound-assisted synthesis was performed using a Transonic 460/H Elma (35 kHz) ultrasound bath.

The bands corresponding to the vibrating bonds of the target compounds were determined by Fourier transform infrared spectroscopy (FTIR, Bruker 27) with the connection of Attenuated Total Reflection (ATR). The crystalline phase composition was determined by X-ray diffraction measurements (XRD Bruker D8 Advance) with CuKα radiation. The surface area of the samples was calculated by the BET equation using the N_2_ adsorption–desorption curves, measured in a Quantachrome Autosorb-1 instrument. The optical absorption of the photocatalysts was determined using a DRS-UV-vis spectrophotometer (Thermo Fisher Scientific-Evolution 600), equipped with an integrating sphere (TFS-Praying Mantis). The morphologies and size of the particles were acquired using FE-SEM (FEI-Nova nanosem 200). The ^29^Si MAS NMR spectra were recorded using a Bruker Advance III 400 MHz, operating at 79.47 MHz, using 4 mm ZrO_2_ rotors, the contact time was 3000 μs, d1 = 4 s with spinning rate of 8 kHz and 8000 scans, and as an internal reference was used talc (−98.1 ppm), the chemical shifts (*δ*) are expressed in ppm.

### General procedure for the SiO_2_–TiO_2_ hybrid catalyst

A set of five molar compositions of SiO_2_–TiO_2_ were evaluated 1 : 4, 1 : 2, 1 : 1, 2 : 1, and 4 : 1. For a 1 : 1 molar ratio, a mixture of TEOS (2083.00 mg), TBOT (3403.20 mg) were placed in an appropriate Erlenmeyer or bottom flask, and 5 mL of distilled water for MH, US, MW, and 10 mL of distilled water for NIR procedures. The mixture was treated using and comparing four different activation modes: near-infrared irradiation during 15 min at 90 °C with vigorous magnetic stirring, placing the magnetic agitator under Flavor-Time; ultrasound bath for 30 min at 25 °C; microwave irradiation during 10 min at 80 °C with 100 W power, and mantle heating for 3 h at 60 °C. For another molar ratio 1 : 2 (1041.50 mg of TEOS and 3403.20 mg of TBOT), 1 : 4 (520.75 mg of TEOS and 3403.20 mg of TBOT), 2 : 1 (4166.00 mg of TEOS and 3403.20 mg of TBOT), and 4 : 1 (4166.00 of TEOS and 1701.60 mg of TBOT) were following the same procedure. Then, the gels were aged and dried for 3 h at 80 °C. Finally, the materials were finely ground and calcinated for 5 h at 500 °C.

### Evaluation of photocatalytic activity (PCA)

The degradations were carried out in a reaction system, consisting of a stainless-steel annular cylinder that serves as a support of four UV light lamps (15 W) with maximum emission at 350 nm. The system has a glass cell of 750 mL of capacity and 8 cm of diameter equipped with a hood of two ports: for the inlet of oxygen, which is supplied by a glass tube whit constant flow 100 mL min^−1^, and the feeding of the sample. The reactor bears a fan placed in the down of the system to avoid heating of the lamps and the reaction mixture, and a magnetic stirring plate to maintain the mix homogeneously.

The PCA of the SiO_2_–TiO_2_ catalysts for the degradation of 4-chlorophenol was evaluated in aqueous solution at pH 6. The catalyst (250 mg) was added a glass cell containing 250 mL of an aqueous solution of 4-chlorophenol (20 ppm). The suspension was magnetically stirred in the dark for 30 min to achieve the adsorption–desorption equilibrium. Then, the UV lights lamps, fan, and oxygen are switch on. The reaction is carried out for 6 h. Then the samples were filtered with 0.22 μg GV millipore membranes, and the solution obtained was stored in the dark for total organic carbon (TOC) analysis by mean of DRS-UV-vis spectroscopy.

## Conclusions

A green approach for the production of silica–titania catalysts, with interesting photocatalytic activity, has been achieved. Compliance with several principles of the Green Chemistry Protocol (1, 3, 5, 6, 10, and 12) has been addressed, with two green features being the employment of non-conventional activation modes with efficacies in the order MW > US > NIR, as opposed to traditional MH (principle 6), and the use of water as a green solvent (principle 5). The prepared catalysts have been extensively characterized by FTIR, XRD, DRS-UV/Vis, BET, FE-SEM, and ^29^Si MAS NMR. The efficacies of the four activation modes have been evaluated and discussed. Finally, the pertinence of the SiO_2_–TiO_2_ catalysts has been suitably demonstrated by evaluating their PCA efficiencies in the degradation of 4-chlorophenol in aqueous solution.

## Conflicts of interest

There are no conflicts to declare.

## Supplementary Material

RA-010-D0RA07569H-s001
