# Supplementary material for: Green and facile sol–gel synthesis of the mesoporous SiO2–TiO2 catalyst by four different activation modes
Source: RSC Adv. 2020 Oct 29;10(65):39580–8. doi: 10.1039/d0ra07569h (PMC9057439; doi:10.1039/d0ra07569h)
Supplement: RA-010-D0RA07569H-s001 [file RA-010-D0RA07569H-s001.pdf]

Electronic Supplementary Material (ESI) for RSC Advances.
This journal is © The Royal Society of Chemistry 2020

Content

1.- Table 1 FTIR data of SiO_2-TiO_2 catalyst, most important bands.

2.- Figure 1 FTIR of SiO_2-TiO_2 for a) 1:4, b) 4:1 molar ratio catalyst obtained by MH, US, NIR and MW.

3.- Figure 2 Diffractograms of SiO_2-TiO_2 for a) 1:1, b) 4:1 molar ratio catalyst.

4.- Table 2 Band gap energy values calculated from DRS-UV-vis spectra.

5.- Figure 3 Curves of Kubelka-Munk of SiO_2-TiO_2 for a) 1:4, b) 4:1 molar ratio catalyst.

6.- Determination of Band gap energy as function of Kubelka-Munk method.

1.-

Table 1 FTIR data of SiO_2-TiO_2 catalyst, most important bands

Bonds	Molar ratio SiO_2-TiO_2 1:2	1:4	4:1	2:1
	[cm^{-1}]			
Si-O-Si	1043-1025	1021-1047	1060-1046	1064-1049
Si-O-Si	801-794	801-798	808-794	794-797
Si-O-Ti	927-905	920-902	952-937	944-939
Ti-O-Ti	436-421	435-416	440-422	435-423

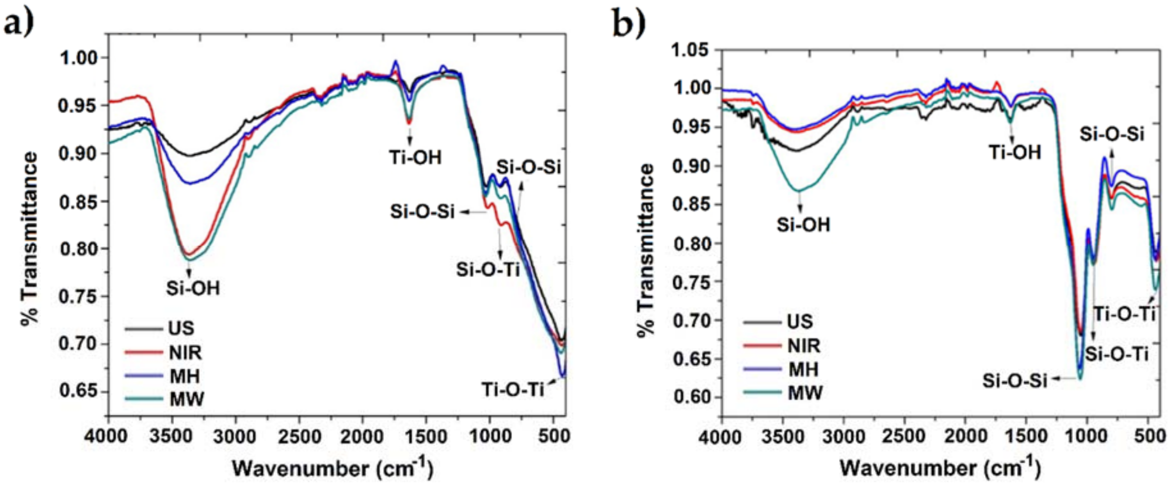

2.- Figure 1 FTIR of SiO_2-TiO_2 for a) 1:4, b) 4:1 molar ratio catalyst obtained by MH, US, NIR and MW.

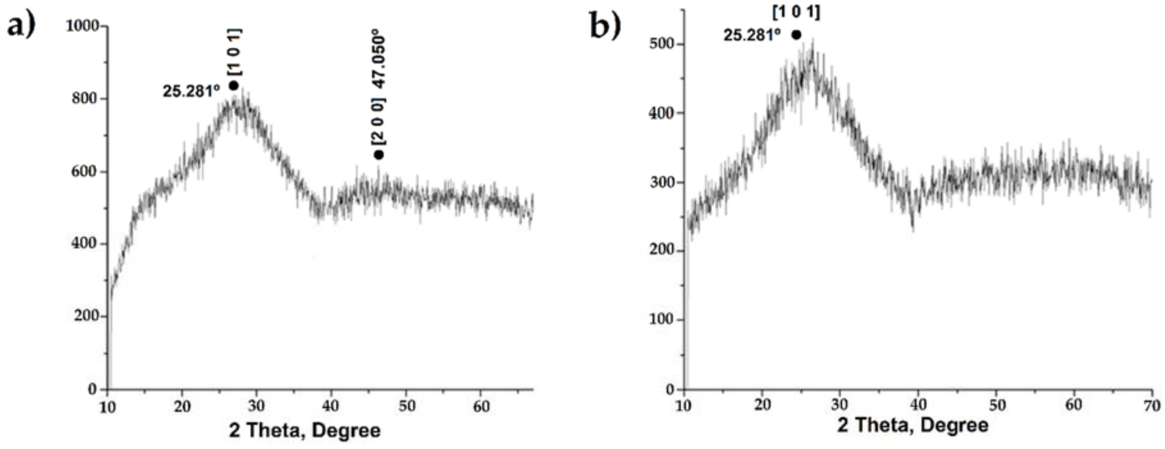

3.- Figure 2 Diffractograms of SiO_2-TiO_2 for a) 1:1, b) 4:1 molar ratio catalyst.

4.-

Table 2 Band gap energy values calculated from DRS-UV-vis spectra

Molar ratio SiO_2-TiO_2	MH	NIR	US	MW
	λ (nm)/Eg (eV)			
**1:1**	368/3.3	363/3.4	378/3.2	375/3.3
**1:2**	380/3.2	364/3.4	372/3.3	370/3.3
**1:4**	377/3.3	375/3.3	381/3.2	382/3.2
**2:1**	360/3.4	359/3.4	358/3.4	361/3.4
**4:1**	355/3.5	360/3.4	366/3.4	361/3.4

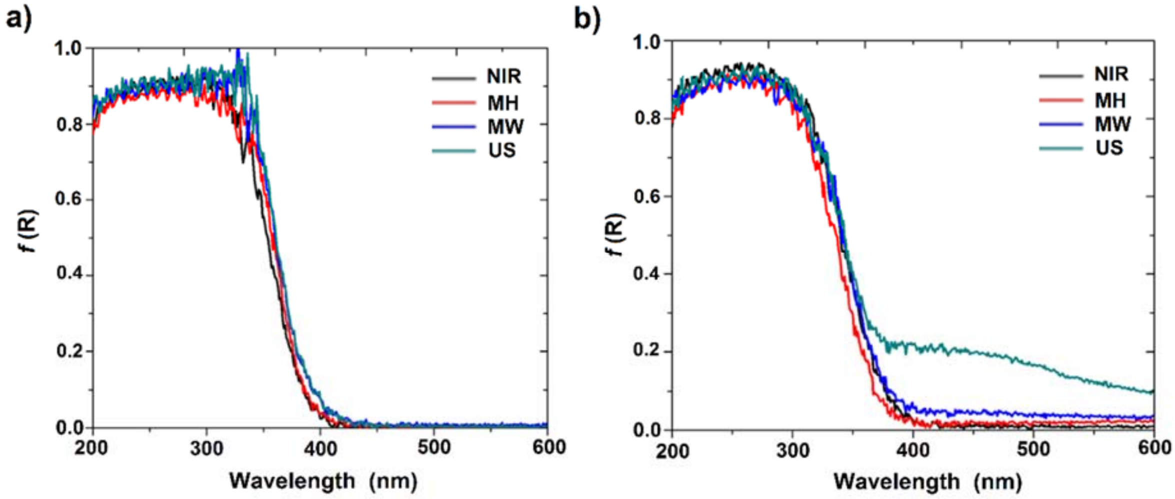

5.- Figure 3 Curves of Kubelka-Munk of SiO_2-TiO_2 for a) 1:4, b) 4:1 molar ratio catalyst.

6.- Determination of band gap energy as function of Kubelka-Munk method.

$$E_g = \frac{hc}{\lambda_g}$$

$$E_g = \frac{(4.09 \text{ x } 10^{-15}\text{eV})\left(3 \text{ x } 10^{17}\,\frac{nm}{s}\right)}{\lambda_g}$$

$$E_g\ (\text{eV}) = \left(\frac{1226.2}{\lambda_g}\right)$$

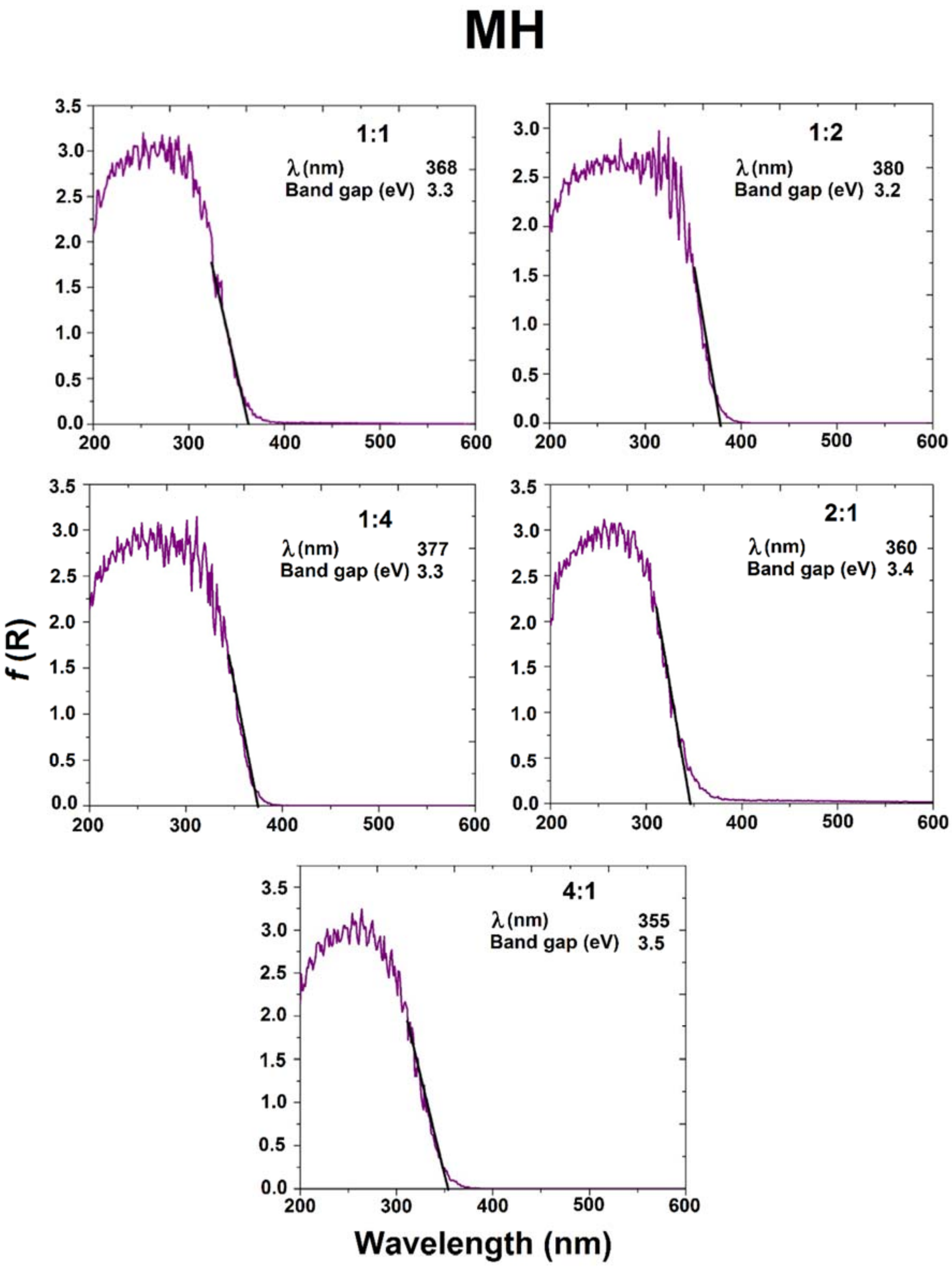
MH
1:1
λ (nm) 368
Band gap (eV) 3.3
1:2
λ (nm) 380
Band gap (eV) 3.2
1:4
λ (nm) 377
Band gap (eV) 3.3
2:1
λ (nm) 360
Band gap (eV) 3.4
4:1
λ (nm) 355
Band gap (eV) 3.5
f (R)
Wavelength (nm)

NIR

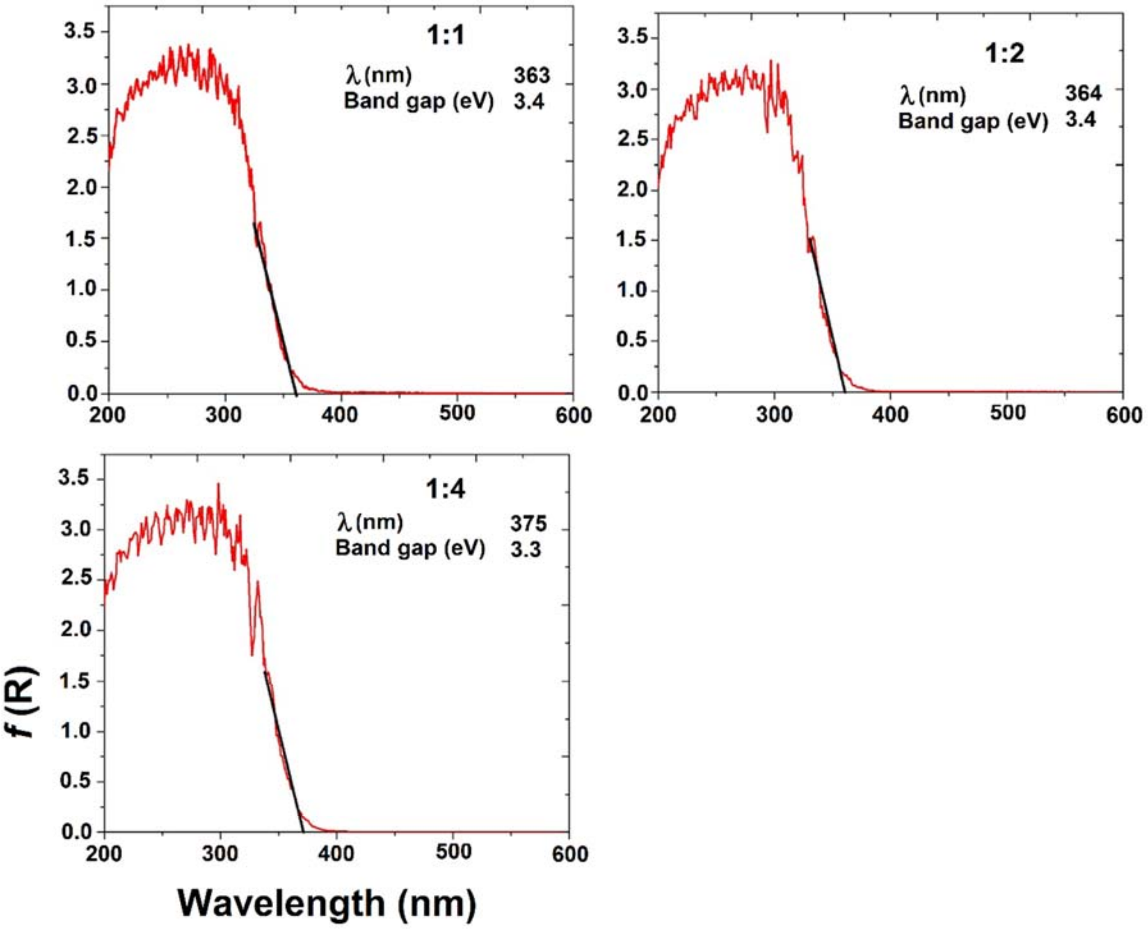
1:1
λ (nm) 363
Band gap (eV) 3.4
1:2
λ (nm) 364
Band gap (eV) 3.4
1:4
λ (nm) 375
Band gap (eV) 3.3
f (R)
Wavelength (nm)
3.5
3.0
2.5
2.0
1.5
1.0
0.5
0.0
200
300
400
500
600

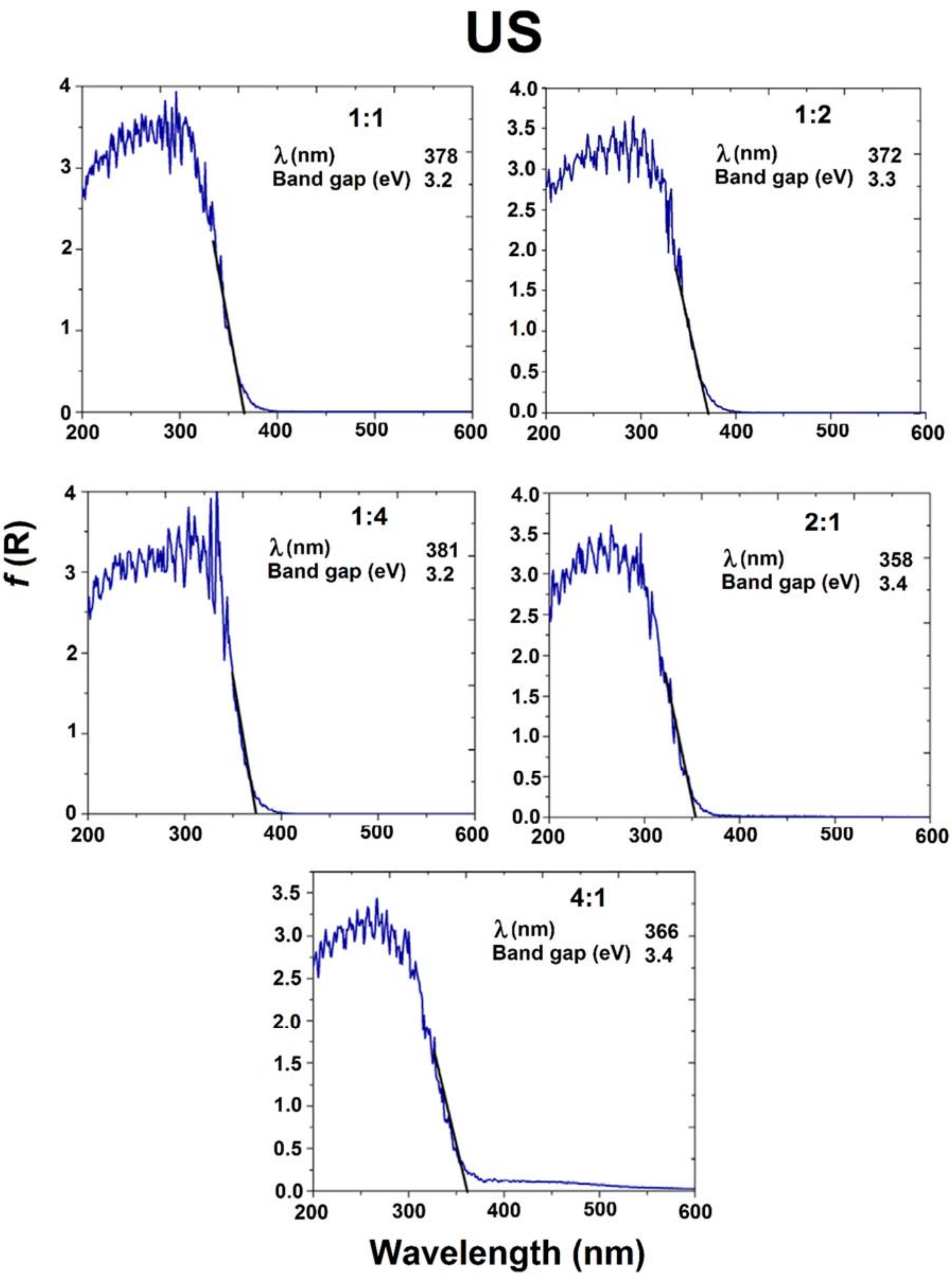

US
1:1
λ (nm) 378
Band gap (eV) 3.2
1:2
λ (nm) 372
Band gap (eV) 3.3
1:4
λ (nm) 381
Band gap (eV) 3.2
2:1
λ (nm) 358
Band gap (eV) 3.4
4:1
λ (nm) 366
Band gap (eV) 3.4
f (R)
Wavelength (nm)

MW

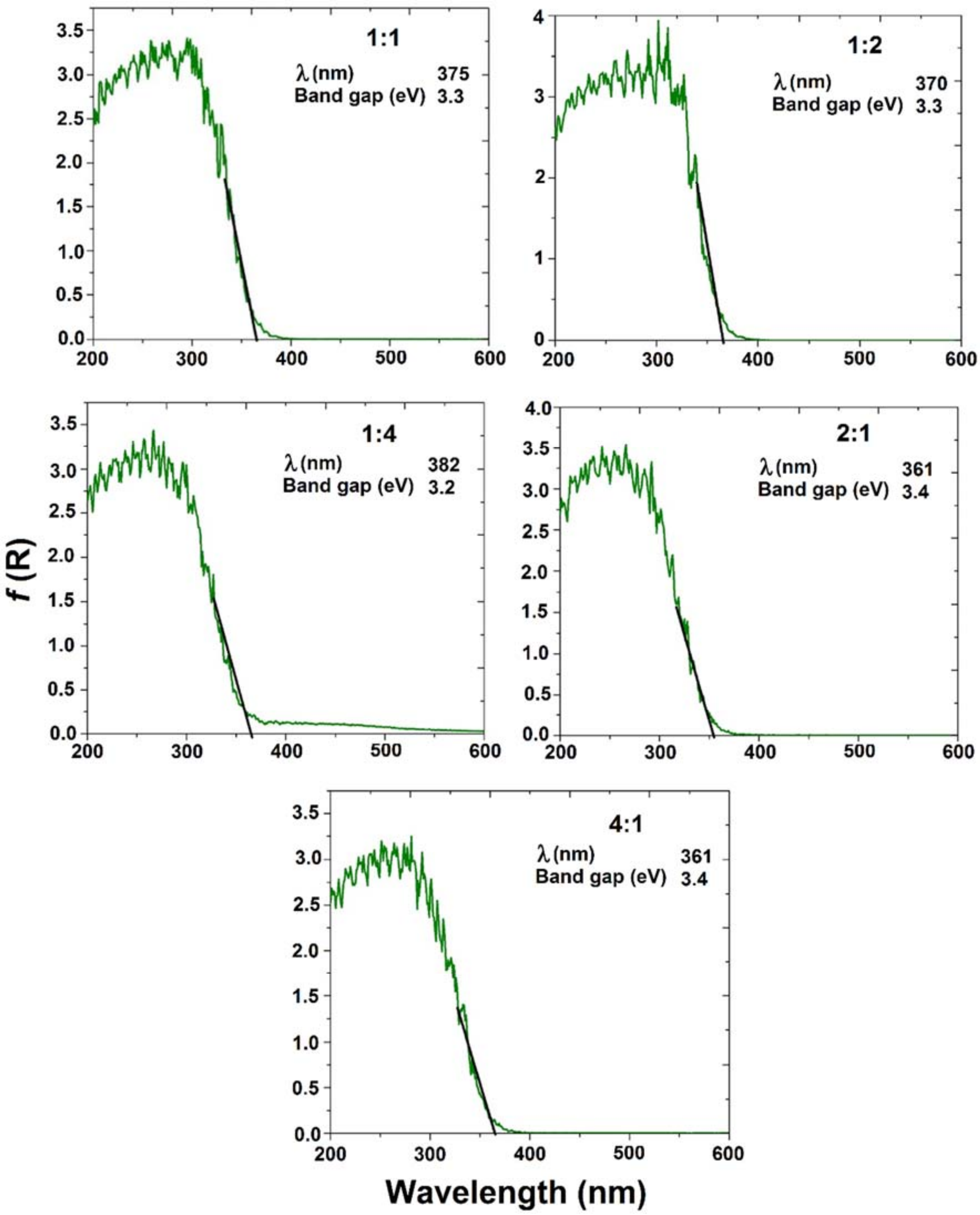
1:1
λ (nm) 375
Band gap (eV) 3.3
1:2
λ (nm) 370
Band gap (eV) 3.3
1:4
λ (nm) 382
Band gap (eV) 3.2
2:1
λ (nm) 361
Band gap (eV) 3.4
4:1
λ (nm) 361
Band gap (eV) 3.4
f (R)
Wavelength (nm)
